# COVID-19 Vaccine Acceptance and Hesitancy among Migrants, Refugees, and Foreign Workers: A Systematic Review and Meta-Analysis

**DOI:** 10.3390/vaccines11061070

**Published:** 2023-06-06

**Authors:** Khalid Hajissa, Hammed-Akanmu Mutiat, Nawal Al Kaabi, Mohammed Alissa, Mohammed Garout, Anood A. Alenezy, Rana H. Almaghrabi, Hayam A. Alrasheed, Maha F. Al-Subaie, Hatem M. Alhani, Ahmad A. Alshehri, Ibrahim Abdullah Almazni, Ali S. Alqahtani, Fayez Saeed Bahwerth, Nourah Hashem Alqethami, Amal A. Alzayer, Ali A. Rabaan

**Affiliations:** 1 Department of Zoology, Faculty of Science and Technology, Omdurman Islamic University, Omdurman 14415, Sudan; 2 Department of Biomedicine, School of Health Sciences, Universiti Sains Malaysia, Kubang Kerian 16150, Kelantan, Malaysia; folasade.mt@gmail.com; 3 Sheikh Khalifa Medical City, Abu Dhabi Health Services Company (SEHA), Abu Dhabi 51900, United Arab Emirates; alkaabin971@gmail.com; 4 College of Medicine and Health Science, Khalifa University, Abu Dhabi 127788, United Arab Emirates; 5 Department of Medical Laboratory Sciences, College of Applied Medical Sciences, Prince Sattam bin Abdulaziz University, Al-Kharj 11942, Saudi Arabia; m.alissa@psau.edu.sa; 6 Department of Community Medicine and Health Care for Pilgrims, Faculty of Medicine, Umm Al-Qura University, Makkah 21955, Saudi Arabia; magarout@uqu.edu.sa; 7 Laboratory Department, Johns Hopkins Aramco Healthcare, Dhahran 31311, Saudi Arabia; anoud.enezy@jhah.com; 8 Collage of Medicine, Dar AlUloom University, Riyadh 13314, Saudi Arabia; 9 Pediatric Department, Prince Sultan Medical Military City, Riyadh 12233, Saudi Arabia; ralmaghrabi@psmmc.med.sa; 10 Department of Pharmacy Practice, College of Pharmacy, Princess Norah Bint Abdulrahman University, Riyadh 11671, Saudi Arabia; haalrasheed@pnu.edu.sa; 11 Pharmacy Department, King Abdullah Bin Abdulaziz University Hospital, Riyadh 11671, Saudi Arabia; 12 Research Center, Dr. Sulaiman Alhabib Medical Group, Riyadh 13328, Saudi Arabia; maha.alsobaie@drsulaimanalhabib.com; 13 Department of Infectious Diseases, Dr. Sulaiman Alhabib Medical Group, Riyadh 13328, Saudi Arabia; 14 College of Medicine, Alfaisal University, Riyadh 11533, Saudi Arabia; 15 Department of Pediatric Infectious Disease, Maternity and Children Hospital, Dammam 31176, Saudi Arabia; hanihm@gmail.com; 16 Department of Infection Control, Maternity and Children Hospital, Dammam 31176, Saudi Arabia; 17 Preventive Medicine and Infection Prevention and Control Department, Directorate of Ministry of Health, Dammam 32245, Saudi Arabia; 18 Department of Clinical Laboratory Sciences, Faculty of Applied Medical Sciences, Najran University, Najran 61441, Saudi Arabia; aaalshehri@nu.edu.sa (A.A.A.); eaalmazni@nu.edu.sa (I.A.A.); 19 Department of Medical Laboratory Sciences, Faculty of Applied Medical Sciences, King Khalid University, Abha 61481, Saudi Arabia; aallmizhr@kku.edu.sa; 20 Laboratory Department, King Faisal Hospital, Makkah 21955, Saudi Arabia; fbahuwayrith@moh.gov.sa (F.S.B.); nhalqethami@moh.gov.sa (N.H.A.); 21 Nursing Department, Erhadah Psychiatric & Mental health, Dammam 31422, Saudi Arabia; 22 Molecular Diagnostic Laboratory, Johns Hopkins Aramco Healthcare, Dhahran 31311, Saudi Arabia; 23 Department of Public Health and Nutrition, The University of Haripur, Haripur 22610, Pakistan

**Keywords:** COVID-19, vaccine, acceptance, hesitancy, rejection, migrants, refugees, foreign workers

## Abstract

Despite the effectiveness of current vaccines in reducing the spread and severity of SARS-CoV-2 infections, many people, including migrants, refugees, and foreign workers, are hesitant to be vaccinated. This systematic review and meta-analysis (SRMA) was conducted to determine the pooled prevalence estimate of the acceptance and hesitancy rates of the COVID-19 vaccine among these populations. A comprehensive search of the peer-reviewed literature indexed in PubMed, Scopus, Science Direct, and Web of Science databases was conducted. Initially, 797 potential records were identified, of which 19 articles met the inclusion criteria. A meta-analysis of proportions using data from 14 studies revealed that the overall acceptance rate of COVID vaccination among 29,152 subjects was 56.7% (95% CI: 44.9–68.5%), while the prevalence of vaccine hesitancy among 26,154 migrants reported in 12 studies was estimated to be 31.7% (95% CI: 44.9–68.5%). The acceptance rate for the COVID-19 vaccination first declined from 77.3% in 2020 to 52.9% in 2021 and then slightly increased to 56.1% in 2022. The most frequent factors influencing vaccine hesitancy were worries about vaccine efficacy and safety. Intensive vaccination campaigns should be implemented to raise vaccination awareness among migrants, which will increase the acceptance rate for the COVID-19 vaccine and result in herd immunity.

## 1. Introduction

The emergence of the COVID-19 pandemic has significantly impacted human society in many aspects, including public health, economic growth, education, and personal well-being [1,2]. Global health has been drastically impacted by the COVID-19 pandemic. By March 29, 2023, over 6.9 million people had died, and nearly 761 million had been infected with SARS-CoV-2 [3]. Despite intensive research efforts, there is currently no effective therapeutic approved for the treatment of SARS-CoV-2 infection. Vaccinating the majority of the world’s population represents a potential breakthrough [4]. Vaccination is currently considered the most powerful and cost-effective measure to combat such a global health crisis. However, vaccination is estimated to be required for approximately 67% of the population to achieve herd immunity and thus stop the pandemic [5]. Therefore, the willingness of the public to be vaccinated is the primary determinant of the success of any mass vaccination program [6].

Despite the approved COVID-19 vaccines being acknowledged to be safe and effective, the public’s confidence and acceptance remain uncertain and changing. A variety of barriers may contribute to the failure of any vaccination program, including inadequate access to healthcare, vaccination costs, inconvenient clinic hours for immunization, and vaccine hesitancy [7]. Vaccine hesitancy, according to the WHO definition, is the delayed acceptance or complete refusal to be vaccinated regardless of vaccine availability. There is a growing concern that vaccine hesitancy is increasing among individuals around the world, affecting limited-, middle-, and high-resource settings [8]. In this regard, scientific reports from a number of countries worldwide investigating attitudes toward prospective COVID-19 vaccines revealed high levels of vaccine hesitancy [9,10].

According to previous SRMA findings, minority ethnic groups were less likely to intend to receive the COVID-19 vaccine [11]. The COVID-19 pandemic has disproportionately affected ethnic minorities, including immigrants, refugees, and asylum seekers, who have higher rates of infection, hospitalization, and death [12,13,14]. In this regard, various studies have highlighted the disproportionate COVID-19 prevalence among migrants, thereby calling for vaccine access to be prioritized among this vulnerable group. Therefore, the International Organization for Migration (IOM) urges governments to include all migrants living in their countries in national COVID-19 vaccination plans, given that no one will be safe until everyone is protected. Furthermore, the success of any vaccine is dependent on both its effectiveness and vaccination coverage. As a result, evaluating the acceptance rate of COVID-19 vaccines is necessary to determine the vaccination status and level of population immunity that will aid in the prevention and control of any pandemic [15]. Accordingly, this SRMA was conducted to address this threat by providing a comprehensive analysis of the existing data to generate reliable estimates of COVID-19 vaccine hesitancy and acceptance among these populations. This will allow public health officials to design vaccination strategies to promote vaccination among this vulnerable group.

## 2. Materials and Methods

### 2.1. Reporting Guidelines

The updated Preferred Reporting Items for Systematic Reviews and Meta-Analyses (PRISMA) was followed when conducting this SRMA (Table S1) [16].

### 2.2. Search Strategies

On 4 September 2020, a comprehensive literature search of peer-reviewed articles indexed in Web of Science, Science Direct, PubMed, and Scopus databases was conducted, and it was later updated on 17 March 2023 to find studies evaluating COVID-19 vaccine acceptance/hesitancy among migrants, refugees, and foreign workers published between January 2020 and March 2023. Furthermore, the reviewed records’ reference lists were manually checked for any additional relevant studies. The comprehensive search strategy for all databases is shown in Table S2.

### 2.3. Selection Criteria

Studies were included in this SRMA if they (1) reported sufficient data to calculate the COVID-19 vaccine acceptance/ hesitancy rate and (2) were conducted among migrants, refugees, and foreign workers irrespective of language restrictions, while articles that did not attempt to determine the prevalence of COVID-19 vaccine acceptance or hesitancy in the target population, as well as those with missing or overlapping data, were excluded. In addition, studies with only abstracts and case reports, as well as review articles, were not included.

### 2.4. Study Selection

The potential studies retrieved from the four electronic databases were managed using the EndNote program, where the duplicated records were removed. Initially, two researchers (H.A.M. and K.H.) independently reviewed the titles and/or the abstracts of the remaining studies. Subsequently, the full texts of all abstracts that appeared to be eligible for inclusion were then obtained. Subsequently, three authors (N.A.A., M.A. and M.G.) carried out full text selection based on the predetermined eligibility criteria. Disagreements between the authors were resolved through discussion.

### 2.5. Data Extraction

Using a standardized data extraction sheet, the relevant data from the potential studies were extracted by four reviewers (A.A., R.H.A., H.A.A. and M.F.A.). The extracted data were then verified by two authors (H.M.A. and A.A.Al.) to minimize errors and ensure consistency. The following information was extracted from each eligible study: the first author’s name, year of publication, target population, study period, host country, acceptance/hesitancy rate, and factors associated with acceptance/hesitancy.

### 2.6. Quality Assessment

The Joana Briggs Institute (JBI) checklist was used by two authors (I.A.A. and A.S.A.) to evaluate the methodological quality (risk of bias) of each study [17]. The two authors’ evaluations were then compared, and any disagreements were resolved through consensus. The quality of studies was either high (with a score of >70% reporting ‘yes’), moderate (with scores ranging from 50% to 70% reporting ‘yes’), or low (scores of <50% reporting ‘yes’) [18,19].

### 2.7. Statistical Analysis

Using random-effects model, the outcomes of the included studies were pooled, while heterogeneity was achieved using Cochran’s Q-test and *I*^2^ statistics. A cut-off value of ≥75% for the *I*^2^ statistic was considered substantial heterogeneity [20], with a *p*-value of < 0.05 indicating a significant degree of heterogeneity. Egger’s regression test and funnel plots were used to identify potential publication bias. Subgroup analysis was carried out to further investigate potential sources of heterogeneity.

## 3. Results

### 3.1. Study Selection

**Figure 1:**
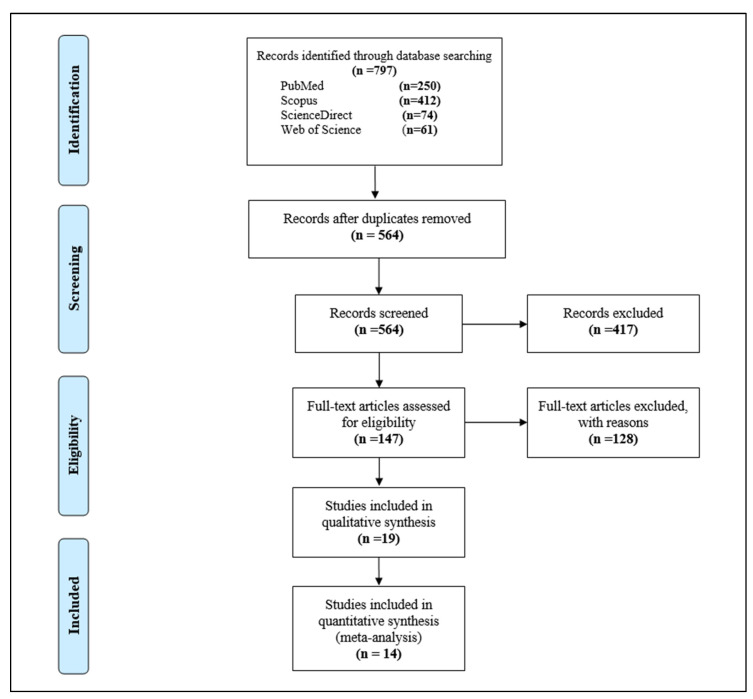
PRISMA flow diagram of study selection.

According to the search strategy described in Table S2, 797 potential studies were retrieved. Of these, 233 articles were duplicates. Another 417 irrelevant studies were excluded during the title and abstract screening. Following full text screening, 128 irrelevant articles were excluded, leaving 19 articles eligible for data extraction and inclusion in the qualitative analysis. Of them, only 14 studies were included in the meta-analysis. A flowchart of the systematic literature search, as well as the selection process, is summarized in the PRISMA format (Figure 1).

### 3.2. Characteristics of Included Studies

The characteristics of all the 19 included studies are presented in Table 1. Three of them were conducted in 2020, eleven in 2021, and the remaining five were conducted between 2020 and 2021 or 2021 and 2022. Overall, 31,537 migrants were included, with sample sizes ranging from 32 in the United Kingdom to 14,917 in China. The migrants were recruited in fourteen different countries across five WHO regions, and one study was carried out in two different regions. The majority of the studies were conducted using online or phone surveys and a cross-sectional design. The acceptance rate was reported in 14 of the 19 included studies, while vaccine hesitancy was reported in 12 of them.

### 3.3. Intention to Accept COVID-19 Vaccine

The primary outcome (COVID-19 vaccine acceptance) was assessed in most of the included studies using the following question: If a COVID-19 vaccine is available, would you get vaccinated? Three options, ‘yes’, ‘no’, and ‘unsure’, were used.

### 3.4. Prevalence of Vaccine Acceptance and Hesitancy

**Figure 2:**
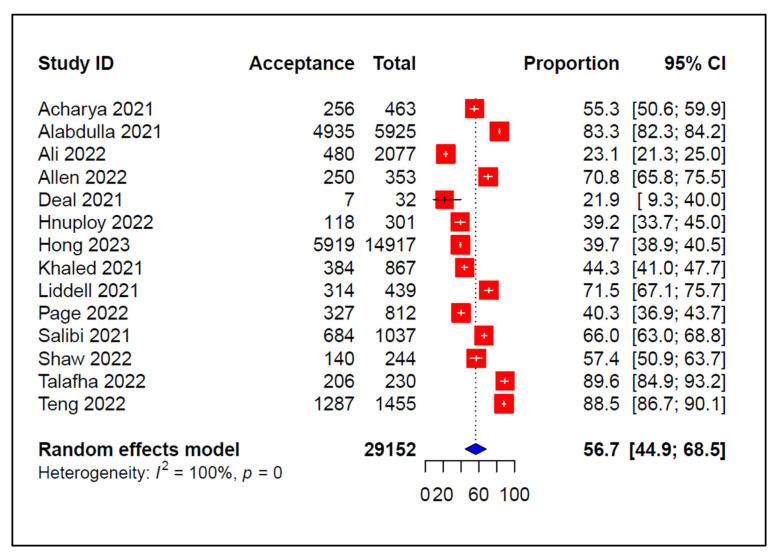
Pooled prevalence of COVID-19 vaccine acceptance [21,22,24,25,27,29,30,31,32,34,35,36,37,38].

**Figure 3:**
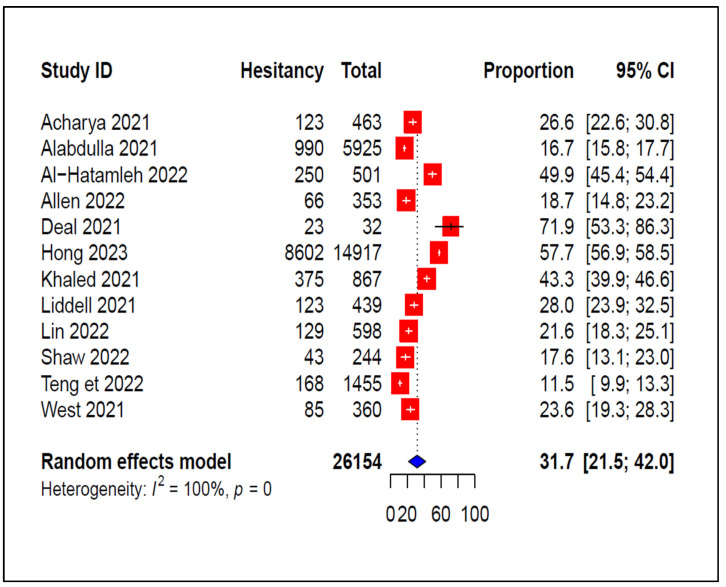
Pooled prevalence of COVID-19 vaccine hesitancy [21,22,23,25,27,29,31,32,33,36,38,39].

The pooled estimate of the COVID-19 vaccination acceptance rate was 56.7% (95% CI: 44.9–68.5%) (Figure 2), with substantial heterogeneity (*I*^2^ = 100%, *p* < 0.0001) observed across the included studies. The highest acceptance rate (88.5 %, 95% CI: 86.7–90.1%) was reported in a study conducted in Japan [38], and the lowest (21.9 %, 95% CI: 9.3–40.0%) was reported in the UK [27]. In contrast, the pooled COVID-19 vaccine hesitancy rate and 95% confidence interval was 31.7 % (95% CI: 21.5–42.0%) (Figure 3). Al-Hatamleh et al. [23] reported the highest hesitancy rate (49.9%, 95% CI: 45.4–54.4%) in a study in Jordan, whereas a study conducted in Japan by Teng et al. [38] reported the lowest vaccination hesitancy rate (11.5% (95% CI: 9.9–13.3%)).

The subgroup analysis for the WHO regions revealed that the region of the Americas had the highest rate of COVID-19 vaccine acceptance (64.3%), followed by the Western Pacific Region (63.7%) and the Eastern Mediterranean Region (61.3%), while the lowest was from the European Region (21.9 %) (Table 2, Figure S1). Based on the participants’ enrolment time, the acceptance rate of COVID-19 vaccination was remarkably high in 2020 (77.3%) before declining to 52.9% in 2021 and then slightly rising to 56.1% in 2022. In contrast, the hesitancy rate increased from 18.7% in 2020 to 29.5% in 2021 and 49.9% in studies conducted between 2021 and 2022. Furthermore, the subgroup analysis revealed that the pooled hesitancy of the COVID-19 vaccine was 71.9% in the WHO region of Europe, 36.5% in the Eastern Mediterranean Region, and 31.0% in the Western Pacific Region (Table 3, Figure S2).

### 3.5. Potential Factors Associated with COVID-19 Vaccine Acceptance and Hesitancy

The most commonly identified factors associated with COVID-19 vaccine acceptance and hesitancy are illustrated in Table 1. The potential side-effects were the main concern for migrants concerning receiving the COVID-19 vaccine. The main concern of migrants in accepting the COVID-19 vaccine was the potential side-effects, which were reported in nine studies [22,23,25,28,31,33,35,38,39]. Vaccine safety [21,22,33,34,37,38], trust [25,26,27,32,33,35], and effectiveness [21,37,38] were also other factors for vaccine hesitancy among migrants. Concerns were also expressed regarding additional key factors, such as the lack of access to the COVID-19 vaccine [27,36], the number of doses [23], and the sufficiency of information [32]. Finally, concerns that the vaccine is religiously prohibited [36], a lack of legal documents [26], and the belief that COVID-19 is not dangerous [28] were also identified as potential factors.

### 3.6. Quality Assessment and Publication Bias

**Figure 4:**
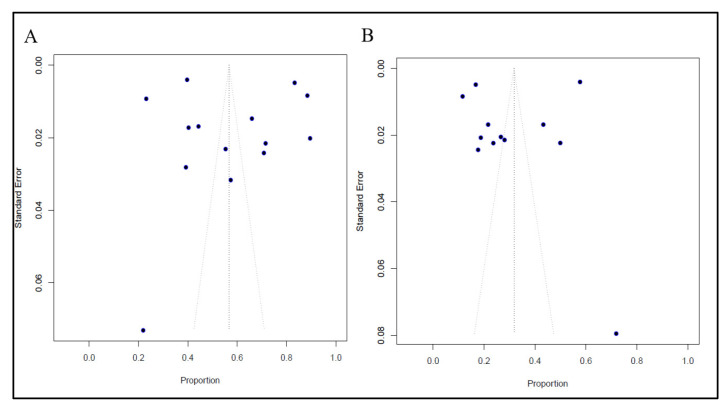
Funnel plot of publication bias of the included studies. (**A**) Vaccine acceptance. (**B**) Vaccine hesitancy.

The methodological quality assessments of all the included study are presented in Table S3. The quality of studies was considered high (low risk of bias) in 10 (52.6%) studies, moderate (moderate risk of bias) in 7 (36.9%) studies, and low (high risk of bias) in 2 (10.5%) studies. The studies were distributed asymmetrically, as seen by a visual examination of the funnel plot (Figure 4), indicating the presence of some publication bias. However, the Egger’s regression test was not statistically significant (*p* > 0.05).

## 4. Discussion

Vaccination remains one of the most cost-effective measures for preventing disease spread and limiting disease burden. Hence, it has played a critical role in containing the COVID-19 pandemic, significantly reducing infection rates, deaths, and the number of serious illnesses associated with COVID-19 infection [40]. However, the vast majority of the adult population should be vaccinated for COVID-19 vaccination strategies to be effective in any country. This goal is unlikely to be met unless all members of the community, including migrants, refugees, and foreign workers, are fully vaccinated. Given the availability of several COVID-19 vaccines, government and public awareness campaigns have convinced many people to get vaccinated. However, vaccine hesitancy remains a major challenge, as many people are still unwilling to receive the vaccination, are less likely to accept the booster shots, and are even hesitant to vaccinate their children. According to this SRMA, the overall prevalence of COVID-19 vaccination acceptance among the 29,152 migrants involved in 14 studies across 14 countries was 56.7% (95% CI: 44.9–68.5%). This rate was lower than previous estimates of COVID-19 vaccine acceptance observed in the global population (67.8%) [41], healthcare workers (65.65%) [42], and healthcare students (68.8%) [43]. The lower acceptance rate among migrants compared to healthcare workers and students is possibly because they may not have been exposed to various health-related information, which may result in low levels of awareness about the COVID-19 vaccines’ safety and effectiveness, thereby influencing their decision to be vaccinated. Meanwhile, healthcare professionals may be well informed about the benefits of vaccination against infectious diseases. In the same manner, the pooled estimate of the acceptance rate in this SRMA was lower than the global average for the general population (67.8%) [41]. It was also lower than the 61% acceptance rate reported in a previous SRMA of 172 studies conducted in 50 countries [44]. Furthermore, Wang et al. estimated that 81.65% of the general population would be willing to receive vaccinations based on the findings of 38 studies [42]. The variations in COVID-19 vaccine acceptance rates could be attributed to study period and study population differences.

Based on our findings, approximately one-third (31.7, 95% CI: 44.9–68.5%) of migrants reported in 12 studies were hesitant to accept a COVID-19 vaccine. The current finding is slightly higher than previous meta-analysis estimates of vaccine hesitancy in the general population, which ranged from 25% to 27.03% [45,46]. In contrast, a higher rate (38.2%) of hesitation to receive COVID-19 vaccination was reported among the general population (38.2%) [47]. The precise underlying factors responsible for COVID-19 vaccination hesitancy among migrants compared to the general population could not be identified. However, the hesitancy, in general, can be associated with several factors, including socioeconomic status, awareness, and knowledge. Nevertheless, a scoping review conducted by Tankwanchi et al. suggested that various migrant populations may have experienced vaccine hesitancy before the onset of the COVID-19 pandemic [48]. In this regard, Polish migrants in the UK were hesitant and reluctant to receive the influenza vaccine [49,50]. Likewise, measles vaccine resistance has been documented among Somali migrants in various Western countries [51,52].

Thus, it is a difficult but crucial task to address the causes of vaccine hesitancy and the barriers to vaccine acceptance. Based on several scientific studies, perceived vaccine safety and effectiveness were the most frequent causes of vaccine hesitancy in previous vaccination programs. However, given that vaccination hesitancy is widely acknowledged to be a complex phenomenon, several predictors other than safety and efficacy concerns were found to be associated with it. This includes the following: high complacency resulted in a more negative perception of disease risks, the negative impact of misinformation and conspiracy beliefs, and access to vaccination services is not very convenient [45,53,54,55].

The subgroup analysis revealed that the regions of America had the highest rate of willingness to receive the vaccine (64.3 %), followed by the Western Pacific Region (63.7%) and the Eastern Mediterranean Region (61.3), while the lowest was from the European Region (21.9 %). The regional variations in acceptance rates have been consistently highlighted in previous reviews [56,57]. This variation can be attributed to concerns about vaccine equity as well as the implementation of different vaccine mandates.

The percentage of people who accept the COVID-19 vaccine showed a declining trend from the beginning of the pandemic, followed by an increasing trend [41]. This was consistent with our findings, as the acceptance rate of COVID-19 vaccination among migrants first declined from 77.3% in 2020 to 52.9% in 2021 and then slightly increased to 56.1% in 2022. Similarly, vaccination intentions have decreased over time, while vaccine hesitancy has increased [58]. Concerns about vaccine safety and efficacy, as well as misinformation about COVID-19, may have contributed to the decline in the acceptance rate at the beginning of the pandemic [42,59]. However, one possible explanation for the increase in vaccine acceptance in 2022 could be the scientific evidence about the COVID-19 vaccine has become more comprehensive.

The reliance on personal research to gather information highlights the important role that social media has in significantly influencing public opinion regarding vaccine uptake. Accordingly, it appears that health communication plans can help people understand and overcome vaccine hesitancy. For example, social media can disseminate reliable information on topics such as the risk of contracting the virus, the severity of the disease, or the effectiveness and safety of the available vaccines. Therefore, healthcare authorities must acknowledge the power and influence of social media and develop creative innovative awareness-raising and information dissemination strategies to boost vaccine uptake.

Although this SRMA used a comprehensive and systematic search strategy, the included studies in this review are from only 14 countries, most of which are developed. In addition, studies conducted in Africa, where nearly one-third of the world’s refugee population resides, are lacking. Thus, future research is required to assess vaccine acceptance/resistance in various studies from Africa and other parts of the world. Such studies could provide an accurate estimate of the true acceptance rate. In addition, the potential determinants of vaccine hesitancy among migrants must be thoroughly investigated. Overall, the pooled estimate of the acceptance and hesitancy rate may not accurately reflect migrants’ intentions to accept COVID-19 vaccination. The direct implications of this SRMA in terms of future research and COVID-19 vaccination policy include the following aspects: First, the review establishes a baseline for future research to provide sufficient detail about COVID-19 vaccine acceptance/ hesitancy among migrants, refugees, and foreign workers. Second, the low vaccination rates act as scientific evidence for decision-makers to increase vaccination awareness, which will increase the acceptance of the COVID-19 vaccine and help control the pandemic.

## 5. Conclusions

The number of migrants who accepted or were willing to accept the COVID-19 vaccine was relatively low, which is a concerning finding that needs be further investigated in future studies. Accordingly, governments and public health authorities should collaborate to implement various vaccination interventions, such as continuing to provide professional training on the effectiveness and safety of the available vaccines in order to improve the COVID-19 vaccine’s acceptance and minimize vaccine hesitancy among migrants, refugees, and foreign workers.

## Figures and Tables

**Table 1:** Major characteristics of the included studies.

Study ID [References]	Country	Study Period	Target Population	Sample Size	Survey Tool	Potential Factors Associated with Acceptance and Hesitancy
Acharya 2021 [21]	South Korea	January and February 2021	Migrants	463	Online survey	Vaccine safety and effectiveness
Alabdulla 2021 [22]	Qatar	15 October and 15 November 2020	Migrants and Qatari nationals	5925	Online survey	Vaccine safety and side-effects
Al-Hatamleh 2022 [23]	Jordan	October 2021 to March 2022	Palestinian refugees	501	Physical interview	Number of doses Experiencing postvaccination adverse effects
Ali 2022 [24]	Lebanon	February, 2021	Refugees	2077	NR	Difficult registration process Security issues
Allen 2022 [25]	USA	July and August 2020	Brazilian immigrant women	353	Online survey	The vaccine had not been fully tested Could have caused serious side-effects or been ineffective Mistrust of vaccines, in general, as well as mistrust of the government and other systems that support vaccine production
Benavides-Melo 2022 [26]	Colombia	August–early September 2021	Venezuelan migrants	926	NR	COVID-19 vaccine is too new Lack of legal documents
Deal 2021 [27]	UK	September 2020 to March 2021	Migrants	32	Physical interview	Lack of trust in the healthcare and vaccination system Lack of access points. Low confidence in COVID-19 vaccines Inadequate knowledge of the vaccine
Führer 2022 [28]	Germany	September 2021 to January 2022	Migrants	204	Online survey	Side-effects and safety Assuming COVID-19 is not dangerous
Hnuploy 2022 [29]	Thailand	October and November 2021	Myanmar migrant workers	301	NR	NR
Hong 2023 [30]	China	2 January to 2 March 2021	Rural-to-urban migrant workers	14,917	Online survey	Factors contributing to the COVID-19 epidemic (lower mortality, infection, and psychological distress) Vaccine factors (decreased vaccination necessity, safety, effectiveness, and reliability)
Khaled 2021 [31]	Qatar	December 2020 to January 2021	Qatari nationals and migrants	867	Phone interview	Concerns about side-effects
Liddell 2021 [32]	Australia	June, 2021	Migrants	439	Online survey	Inadequate information about the vaccine and its effects Logistical barriers (waiting times, concern that the vaccine will be expensive) Trust barriers
Lin 2022 [33]	Canada	15–21 June 2020	Im/migrants	598	Online survey	Vaccine safety Side-effects and mistrust
Page 2022 [34]	USA, Switzerland, Italy, and France	February–May 2021	Undocumented migrants	812	NR	Vaccine safety
Salibi 2021 [35]	Lebanon	January–February 2021	Syrian refugees	1037	Phone interview	Newness of the vaccine Belief that the vaccine is not essential Concern over the vaccine’s side-effects, interactions with other drugs, and lack of trust in the system
Shaw 2022 [36]	USA	December 2020 March 2021	Refugees	244	Verbal survey	Concerns that the vaccine is religiously prohibited Access to vaccination
Talafha 2022 [37]	Jordan	January to March 2022	Refugees	230	Web-based study	Safety and effectiveness
Teng 2022 [38]	Japan	October, 2021	Migrants	1455	Internet survey	Side-effects Vaccine’s safety and efficacy
West 2021 [39]	Bangladesh	(January/February 2021)	Foreign workers	360	Phone interview	Side-effects of the vaccine

Key: UK: United Kingdom, USA: United States of America, NR: not reported.

**Table 2:** Pooled estimates of COVID-19 vaccine acceptance in different subgroups.

Subgroups	Acceptance Rate [95% CIs] (%)	Number of Studies Analyzed	Total Number of Subjects	Heterogeneity	
				*I^2^*	*p*-Value
Total	56.7 [44.9; 68.5]	14	29,152	100%	<0.0001
WHO regions					
Western Pacific Region	63.7 [43.1; 84.4]	4	17,274	100%	<0.0001
Eastern Mediterranean Region	61.3 [37.0; 85.4]	5	10,136	100%	<0.0001
Region of the Americas	64.3 [51.1; 77.4]	2	597	91%	< 0.01
European Region	21.9 [9.3; 40.0]	1	32	NA	NA
South-East Asia Region	39.2 [33.7; 45.0]	1	301	NA	NA
Region of the Americas and European Region	40.3 [36.9; 43.7]	1	812	NA	NA
Survey Tool					
Online survey	65.2 [48.5; 81.9]	8	25,859	100%	<0.0001
Physical interview	40.2 [5.5; 75.0]	2	276	95%	< 0.01
Phone interview	55.1 [33.9; 76.4]	2	1904	99%	< 0.01
Enrolment Time					
2020	77.3 [ 0.0; 49.7]	2	6278	96%	<0.01
2021	52.9 [38.1; 67.8]	8	21,501	100%	<0.0001
2020–2021	42.1 [22.7; 61.5]	3	1143	92%	< 0.01
2022	56.1 [44.9; 68.5]	1	230	NA	NA

CIs: confidence intervals; NA: not applicable.

**Table 3:** Pooled estimates of COVID-19 vaccine hesitancy in different subgroups.

Subgroups	Hesitancy Rate [95% CIs] (%)	Number of Studies Analyzed	Total Number of Subjects	Heterogeneity	
				*I^2^*	*p*-Value
Total	31.7 [21.5; 42.0]	12	26,154	100%	<0.0001
WHO regions					
Western Pacific Region	31.0 [12.0; 50.0]	4	17,274	100%	<0.0001
Eastern Mediterranean Region	36.5 [16.6; 56.5]	3	7293	100%	<0.01
Region of the Americas	19.7 [17.2; 22.2]	3	1195	9%	0.33
European Region	71.9 [53.3; 86.3]	1	32	NA	NA
South-East Asia Region	23.6 [19.3; 28.3]	1	360	NA	NA
Survey tool					
Online survey	25.8 [14.5; 37.2]	7	24,150	100%	<0.0001
Physical interview	45.8 [15.2; 76.4]	3	777	98%	<0.01
Phone interview	33.5 [14.2; 52.7]	2	1227	98%	<0.01
Enrolment time					
2020	18.7 [15.7; 21.7]	3	6876	76%	0.02
2021	29.5 [14.5; 44.6]	5	17,634	100%	<0.0001
2020–2021	43.5 [13.3; 73.7]	3	1143	98%	<0.01
2021–2022	49.9 [45.4; 54.4]	1	250	NA	NA

CIs: confidence intervals; NA: not applicable.

## Data Availability

The data generated in this study are available within the manuscript and Supplementary Materials.

## Supplementary Materials

The following supporting information can be downloaded at: https://www.mdpi.com/article/10.3390/vaccines11061070/s1, Table S1: PRISMA checklist. Table S2: Detailed search strategy. Table S3: Quality assessment of the included studies. Subgroup analysis representing the COVID-19 vaccine acceptance. Figure S1: Subgroup analysis representing the COVID-19 vaccine acceptance. Figure S2: Subgroup analysis representing the COVID-19 vaccine hesitancy based on: A. WHO regions, B. Survey tool and C. Enrolment time.
